# Exercise, exerkines, and cardiometabolic health: from individual players to a team sport

**DOI:** 10.1172/JCI168121

**Published:** 2023-06-01

**Authors:** Jeremy M. Robbins, Robert E. Gerszten

**Affiliations:** 1 Division of Cardiovascular Medicine and; 2 CardioVascular Institute, Beth Israel Deaconess Medical Center, Boston, Massachusetts, USA.

## Abstract

Exercise confers numerous salutary effects that extend beyond individual organ systems to provide systemic health benefits. Here, we discuss the role of exercise in cardiovascular health. We summarize major findings from human exercise studies in cardiometabolic disease. We next describe our current understanding of cardiac-specific substrate metabolism that occurs with acute exercise and in response to exercise training. We subsequently focus on exercise-stimulated circulating biochemicals (“exerkines”) as a paradigm for understanding the global health circuitry of exercise, and discuss important concepts in this emerging field before highlighting exerkines relevant in cardiovascular health and disease. Finally, this Review identifies gaps that remain in the field of exercise science and opportunities that exist to translate biologic insights into human health improvement.

## Introduction

Regular exercise remains one of the bedrocks of human health promotion, benefiting cognition, immune and neurohormonal networks, and the musculoskeletal and cardiovascular systems. The multiorgan effects of regular exercise — including but not limited to improved cardiorespiratory fitness (CRF) — prevent and combat a broad spectrum of human diseases, including cancer, cardiometabolic disease, and frailty, and promote longevity (1–5). These benefits are juxtaposed with the pandemics of sedentary behavior, obesity, and type 2 diabetes mellitus (T2D), all of which have been exacerbated by the COVID-19 pandemic (6). It is understandable, then, that unraveling the biochemical pathways and molecular biology that underlie exercise and its salutary effects remains a major focus of the health community (7).

Here we summarize major insights from human studies demonstrating the benefits of regular exercise and improved CRF for cardiometabolic health. We describe the regulation of substrate metabolism required across the spectrum of exercise, detailing the cardiac-specific substrate metabolism that underlies acute exercise and adaptations to exercise training. With this as background, we focus in particular on the emerging field of “exerkines,” and highlight exerkines relevant in cardiovascular disease. We subsequently discuss the challenges and knowledge gaps that remain in the field, as well as opportunities to translate the increasingly vast molecular information into knowledge that can improve human health.

## Exercise in prevention and treatment of cardiometabolic disease

The growing epidemics of obesity, T2D, nonalcoholic fatty liver disease, and their downstream sequelae have highlighted the urgent need to decrease sedentary behavior and increase physical activity. Regular exercise — a broad term reflecting structured, purposeful physical activity (8) — has proven effective in the prevention and treatment of cardiometabolic diseases. Understanding the evidence in existing human studies, as well as current gaps, is critical to addressing the burden of metabolic diseases.

### Obesity and metabolic syndrome.

Changes in weight are influenced by total energy expenditure — itself determined by resting energy expenditure and the thermic effects of feeding and physical activity. Thus, the precise impacts of regular exercise or exercise training interventions on weight loss in obesity have been difficult to determine, at least in part due to variability in diets across studies. Clinical trials of supervised, mostly aerobic exercise training have demonstrated either reductions in body mass and/or improvements in body composition among overweight or obese adults maintaining their current diet (9, 10); however, these trials demonstrated high rates of attrition, limiting the generalizability of findings. Lifestyle interventions that have included dietary interventions in addition to regular exercise have shown greater effects on weight loss than exercise in isolation (11–13). A more contemporary exercise dose-response trial in abdominally obese White Canadian subjects showed that exercise volumes — but not intensities — influenced weight loss, whereas benefits in maximal oxygen uptake (VO_2_max) and glucose homeostasis were seen in those undergoing high-intensity interventions (14). These findings point to the limitations of focusing on isolated health outcomes and the importance of recognizing the pleiotropic effects of regular exercise.

### Type 2 diabetes.

Several lines of evidence point to the benefits of increased physical activity and regular exercise in the prevention of T2D as well as its treatment. In two seminal randomized trials of lifestyle interventions that combined regular physical activity and dietary counseling in participants at high risk for T2D, there was up to a 58% lower risk of developing diabetes in the lifestyle arms at 3 to 4 years (15, 16). In individuals with established T2D, both aerobic training and resistance training improve measures of glucose and insulin homeostasis and/or cardiometabolic risk factors; however, combined training may provide greater benefits than either modality alone (17–19). Indeed, the American College of Sports Medicine and the American Diabetes Association have adopted recommendations for combined training among individuals with T2D (20). While studies examining the impact of high-intensity interval exercise training in individuals with T2D have demonstrated promising findings in regard to glycemic control, body composition, and even myocardial function (21–23), the potential for musculoskeletal injuries raises caution about its widespread use in this population (24). It should be noted that weight loss — as an effect of increased physical activity and/or exercise — remains a critical mediator of the cardiometabolic benefits in people with T2D (20).

### Cardiovascular disease.

The modern-day study of exercise and CVD dates back to observations by Jeremy Morris in the mid–20th century that coronary heart disease risk differed among sedentary bus drivers versus non-sedentary ticket collectors (25). Since then, a wealth of information regarding the effects of physical activity and exercise on CVD has been produced. Dedicated reviews on the prevention and treatment of atherosclerotic CVD (ASCVD) (3, 8, 26) and heart failure (27, 28) exist, and an expanded discussion of these topics is beyond the scope of this Review. However, specific areas of uncertainty exist regarding exercise and CVD, including the potential for a U-shaped relationship between exercise volume and all-cause mortality among those with established CVD (29); the pathogenesis and clinical relevance of coronary artery calcification seen in athletes or those who participate in high-volume exercise (30); and the type of training modality that is most beneficial in CVD, particularly in heart failure with preserved ejection fraction (31).

Notwithstanding these areas of uncertainty, the wealth of data supports the fact that regular exercise is generally beneficial among individuals with ASCVD and heart failure. In contrast, the relationship between increased physical activity and CRF, exercise, and cardiac arrhythmias, including atrial fibrillation (AF), is less clear. Individuals with higher levels of CRF have lower risk of both incident AF and AF burden (32, 33), and this relationship may be modifiable by exercise training (34) — in other words, improvements in CRF may be associated with lower risk of developing AF, although additional data from a small clinical trial of exercise training show no change in this relationship (35). In contrast, numerous observational studies have demonstrated that athletes and those performing high-volume and high-intensity exercise have higher rates of incident AF than non-athletes or those performing moderate-intensity exercise (36–40). A more recent nonlinear meta-regression analysis showed that while moderate-volume exercise (5–20 metabolic equivalent of task [MET] hours per week, reflecting the energy cost relative to the resting metabolic rate) was protective against AF risk, higher volumes (>20 MET h/wk) were unrelated to AF risk, and those with very high volumes had a trend toward increased risk (41). Taken together, these data support a potential J-shaped relationship that has been acknowledged by a recent scientific statement by the American Heart Association (29).

Our understanding of the relationship between exercise and aortic disease is more limited, with cross-sectional data suggesting that high volumes of endurance exercise are associated with thoracic aortic dilatation (42). Whether this finding is pathologic or a physiologic adaptation similar to “athlete’s heart” remains uncertain, and longitudinal data will be needed to help understand its clinical implications.

### Cardiorespiratory fitness.

Perhaps no concept so clearly conveys the value of physical activity and regular exercise to human health as Charles Darwin’s “survival of the fittest” (43). While evolutionary pressures have varied considerably, contemporary studies have so consistently demonstrated a strong and independent inverse relationship between CRF and mortality (44–46) that major cardiovascular and preventive health groups have called for its establishment as a clinical vital sign (47). Both cardiac and ventilatory systems effectively define the limits of CRF; however, the performance of muscular work is inextricably linked to the efficient coupling of metabolism to these systems (48). Thus, efforts to understand how exercise training imparts favorable metabolic changes may in turn provide insights into human fitness and longevity.

## Exercise substrate metabolism and the heart

**Figure 1:**
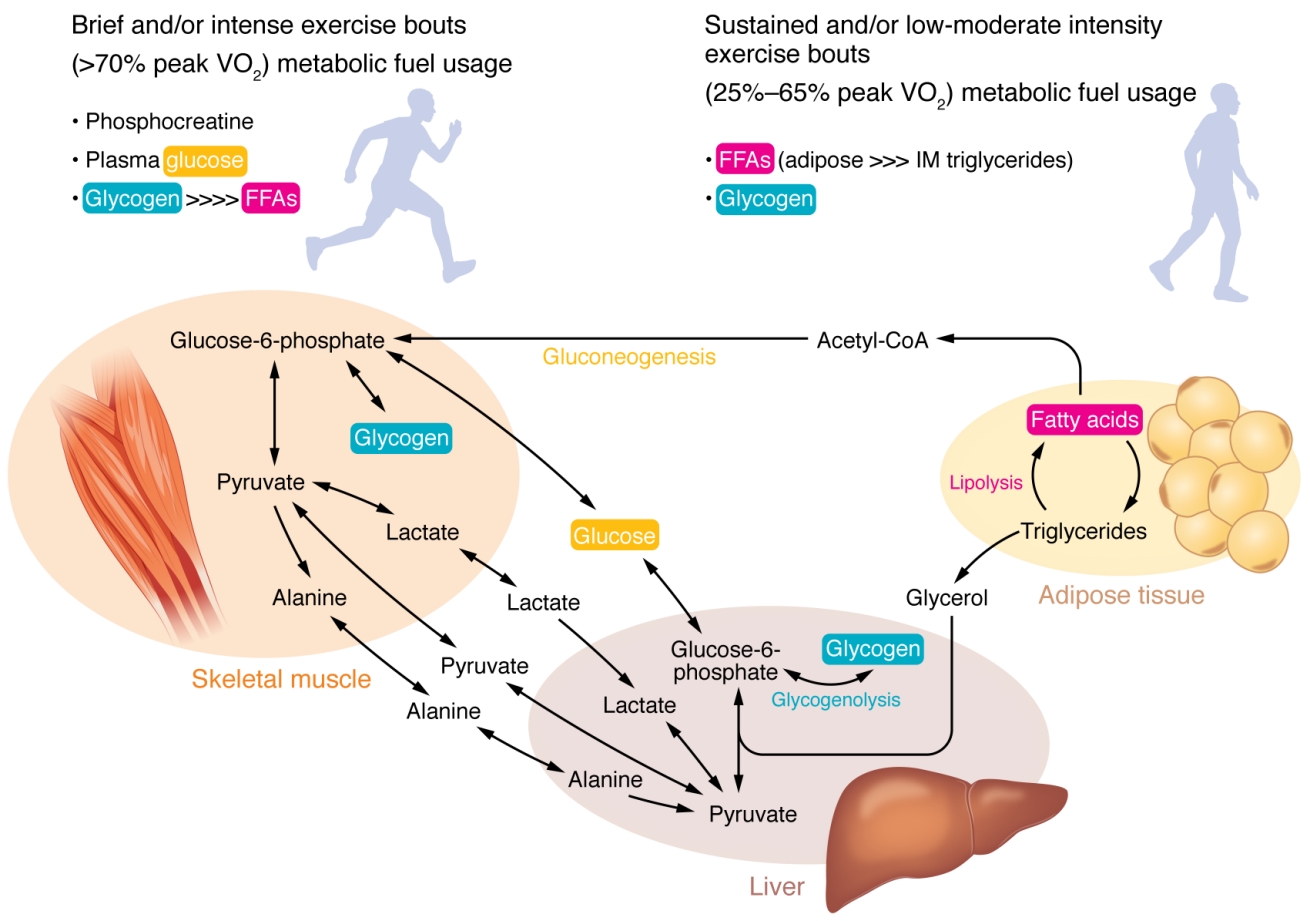
Substrate metabolism and fuel sources according to exercise intensity and duration. Exercise stimulates lipolysis and liberates free fatty acids (FFAs) that become fuel sources for the working skeletal muscle. Similarly, hepatic glycogenolysis and gluconeogenesis promote fuel substrates for the working muscle, and gluconeogenesis helps recycle metabolic end products of exercise (e.g., lactate, alanine, pyruvate). Skeletal muscle itself contains glycogen. Short and maximal-power activity is preferentially fueled by anaerobic metabolism phosphocreatine stores, which are quickly depleted, and subsequent glucose and glycogen stores, whereas sustained activity at lower intensities is primarily fueled by fatty acid oxidation.

The ability to perform locomotor activity is wholly dependent on the skeletal muscle receiving a constant supply of adenosine triphosphate (ATP) for fuel. The body’s ability to make ATP available under conditions ranging from maximal-power-generating bursts to prolonged endurance exercise highlights its remarkable metabolic plasticity. Numerous dedicated reviews have described the processes that govern exercise metabolism and its adaptations to training (49–54). Notably, these reviews emphasize the central roles that skeletal muscle, liver, and adipose tissue play in maintaining metabolic homeostasis during exercise perturbations (Figure 1). In addition to these organs, the heart’s ability to deliver a steady supply of oxygen and nutrients to end organs during acute exercise, as well as adapt in response to the demands of chronic exercise, showcases its metabolic flexibility and highlights biochemical pathways that have generated interest for their therapeutic potential.

### Cardiac metabolism: acute exercise.

The human heart and cardiomyocytes are unique in that they must couple ATP production to very high rates of turnover in order to meet the demands of a muscle that incessantly contracts. Failure to meet these demands results in systemic structural and functional impairments collectively known as “heart failure”; thus, the heart can be considered the most metabolically demanding organ (55). Acute exercise demonstrates the extremes of this process; during intense exercise, cardiac output can increase between 5- and 8-fold, and marked functional hyperemia occurs such that myocardial oxygen consumption (MV_O2_) may increase up to 10-fold (56–58). While fatty acids are the dominant fuel substrate in the healthy heart under resting conditions, the myocardium utilizes a variety of energy sources, including carbohydrates, amino acids, ketone bodies, and lactate depending on their availability and the physiologic state, leading to its characterization as an “omnivore” (59–61). During acute, moderate- to high-intensity exercise, human and rodent studies have shown that lactate and non-esterified fatty acids are the heart’s predominant respiratory substrates (62, 63), with myocardial glucose oxidation increasing during moderate-intensity exercise (30%–55% VO_2_max) and decreasing during high-intensity exercise (75% VO_2_max) (62, 64). While lactate suppresses adipose tissue lipolysis and subsequent release of plasma free fatty acids during exercise through its binding to hydroxycarboxylic acid receptor 1 (HCAR-1) (65), it may enhance myocardial fatty oxidation (66, 67). The contribution of lactate oxidation to overall myocardial oxidation is modestly increased during low- to moderate-intensity exercise (62); however, lactate becomes the dominant myocardial substrate under conditions of high-intensity exercise, when arterial lactate levels can rise 10-fold (68, 69). In studies of isolated perfused rat hearts, glycogen oxidation is suppressed and glycogenolysis attenuated under conditions that mimic high-intensity exercise (i.e. high lactate levels) (66).

Since Randle et al. (70) first described the tight regulatory control of glucose oxidation by the presence of fatty acids (the glucose–fatty acid cycle, or Randle cycle) decades ago, new insights have helped reveal the molecular mechanisms that allow the heart such enormous metabolic flexibility, including allosteric control of regulatory enzymes, transcriptional and posttranslational modification, and the local availability and effects of substrates and their by-products. These processes have been reviewed elsewhere in detail (71, 72).

In addition to the pathways that govern the Randle cycle, the serine-threonine kinase 5′-AMP–activated protein kinase (AMPK) has emerged as an important metabolic regulator in the heart. AMPK has been described as a cellular energy gauge — stimulating fatty acid and glucose oxidation and glycolysis and inhibiting energy-consuming biosynthetic processes under conditions of high AMP/ATP ratios (73). Rat and murine studies have shown that graded increases in AMPK activation during acute exercise are associated with GLUT4 translocation to the sarcolemma, and that glucose uptake and glycolysis failed to increase under conditions of myocardial ischemia in transgenic mice expressing a kinase-dead (K47R) AMPK, despite their exhibiting a normal resting phenotype (74, 75). AMPK inhibits anabolic activities such as protein synthesis through its effects on mTOR/p70S6K and eEF2 (76, 77); more recently, investigators showed that AMPK activation prevented cardiac hypertrophy by inhibiting O-GlcNAcylation (78), though conflicting data exist in an O-GlcNAc transferase–knockout mouse model that did not modulate AMPK activation (79). Taken together, these studies support an important role for AMPK in regulating myocardial metabolism, although questions remain about the varying AMPK substrates and mechanistic effects in different pathophysiologic states (80).

### Cardiac metabolic adaptations: response to training.

Beginning with the landmark Dallas Bed Rest and Training Study, performed in 1966, numerous human studies have helped characterize the cardiac structural and functional adaptations that occur with exercise training (81–85). A central dogma from exercise training studies is that the type (dynamic versus static) and intensity of exercise largely determine the nature of cardiac adaptation. Dynamic (aerobic or endurance) exercise promotes eccentric left ventricular hypertrophy — with cardiomyocyte sarcomeres being added in series — as well as right ventricular dilation, both changes occurring with preserved systolic and even enhanced diastolic function. In contrast, static (isometric or strength training) exercise elicits concentric remodeling with sarcomeres added in parallel (86–88).

Importantly, several changes in cardiac metabolism occur in response to regular exercise, and studies contrasting physiologic versus pathologic cardiac hypertrophy have greatly informed our understanding of the biochemical pathways that underlie them (89, 90). While mixed data exist, endurance exercise training consistently enhances fatty acid oxidation in cardiomyocytes, in part through the activation of PPARγ coactivator-1α (PGC-1α) (91, 92), a master regulator of mitochondrial energetics in skeletal muscle and the heart (93, 94). Increased fatty acid oxidation rates and enhanced mitochondrial biogenic responses in exercise-trained hearts contrast with the reduced oxidative capacity seen in pathologic cardiac conditions (95, 96). Murine studies have shown that endurance exercise training promotes mitochondrial biogenesis, in part, through an endothelial nitric oxide synthase–dependent (eNOS-dependent) pathway (97). Limited but notable data support the concept that exercise intensity influences metabolic changes in the heart and reinforce the importance of specifying the exercise exposure when describing exercise effects. For example, one study showed that C57BL/6J mice undergoing 10 weeks of high-intensity interval training (HIIT) demonstrated a marked increase in glucose oxidation and a relative decrease in fatty acid oxidation, findings not seen in mice undergoing moderate-intensity training (98). In contrast to studies of skeletal muscle, only HIIT mice demonstrated increased myocardial citrate synthase activity.

Despite advances in our understanding of the metabolic changes that occur with exercise training, key questions remain about whether these biochemical changes are cause or consequence of the cardiac structural or functional adaptations in the heart. Riehle and colleagues used a swim training mouse model to show how insulin and insulin-like growth factor 1 (IGF-1) signaling mediates physiologic hypertrophy but also metabolic adaptations after endurance training (99). Through a cardiomyocyte-specific deletion in insulin receptor substrate 1 (IRS1) and IRS2 (referred to as CIRS1 and CIRS1 knockouts, respectively), the authors showed that hypertrophic and mitochondrial adaptations to training were attenuated in both knockout models, with impaired Akt signaling and IRS isoform–specific effects on PGC-1α levels seen. Interestingly, the same group recently demonstrated the role of IRS1/Akt signaling in mediating cardiac remodeling in a murine pressure overload (transverse aortic constriction) model (100). Gibb et al. showed that modulation of glycolysis can impart both cardiac structural and functional changes that recapitulate those seen with endurance exercise training using mouse models that exhibit high or low phosphofructokinase type 2 (PFK2) enzymatic activity in the heart (Glyco^Hi^ and Glyco^Lo^, respectively). As PFK2 is a central regulator of glycolysis through allosteric control of the enzyme PFK1, the authors showed that low PFK1 activity through expression of a kinase-dead PFK2 transgene (Glyco^Lo^ hearts) recapitulated physiologic cardiac hypertrophy — including a similar gene expression profile (101). More recently, rat cardiomyocyte and in vivo studies demonstrated that glucose consumption and subsequent higher levels of intracellular aspartate as a nitrogen donor for nucleotide synthesis were necessary to promote cardiac hypertrophy (102). Other proteins and small-molecule metabolites have been proposed as metabolic signaling molecules in the hypertrophic response, although the mechanistic links and specific relevance to exercise training remain unclear (103).

## Exercise crosstalk: the role of exerkines

**Figure 2:**
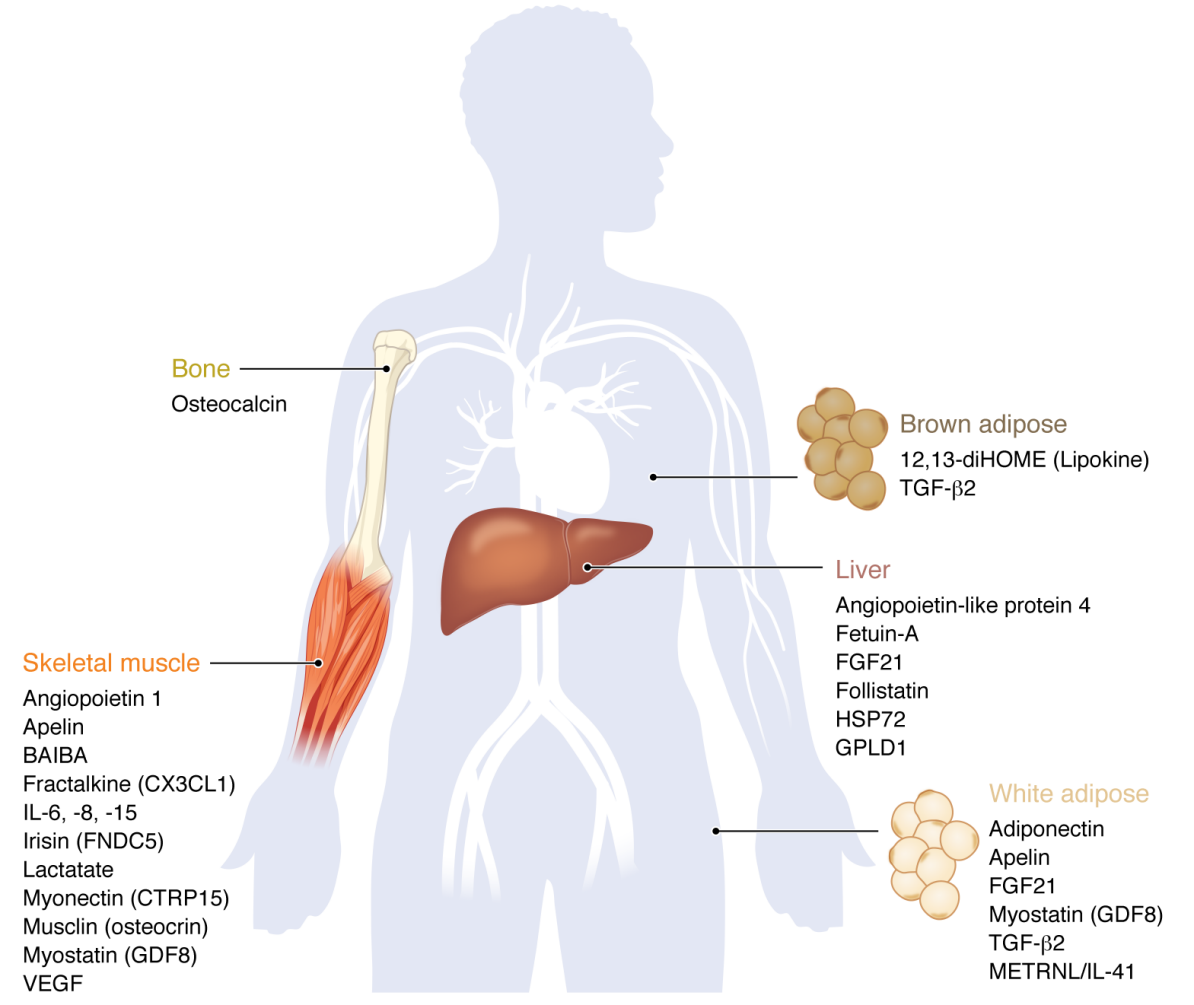
Exerkines according to primary tissue source. Exercise-induced secreted and bioactive factors according to primary tissue origin. Note that several exerkines have multiple tissue sources.

Reductionist approaches to studying exercise metabolism have generated a wealth of knowledge regarding individual organs but, by their nature, have yielded fewer insights into how health benefits are communicated at the global level of an organism. The increased recognition of exerkines (104) — signaling molecules that are released by tissues into the circulation in response to exercise stimuli and impart health effects through local (autocrine or paracrine) or distant (endocrine) means — serves as a paradigm to help better understand the interorgan communication that takes place in response to exercise. While this concept has long been recognized, much of the focus has centered on muscle-secreted factors (myokines), beginning with the identification of interleukin-6 (IL-6) as a metabolic signaling molecule (105, 106). Over the past two decades, emerging high-throughput molecular profiling technologies have further enabled the identification of small-molecule metabolites, lipids, peptides, and nucleic acids that come from adipose tissue (adipokines), liver (hepatokines), bone (osteokines), and the nervous system (neurokines) in addition to myokines (Figure 2). Despite this, the exact tissue sources of many of the exercise-induced plasma biochemicals remain uncertain (107, 108), although efforts are being made to help localize exerkines (109). The topic of exerkines has been reviewed recently (110); however, we further expand upon questions regarding their temporal effects and modes of transport into circulation, before focusing on cardiovascular-specific exerkines.

### Temporal effects: acute versus chronic exercise stimuli.

An important consideration in the field is the temporal relationship between exercise bouts, training, and exerkine effects. While the acute and chronic effects of exercise-induced transcription at the skeletal muscle level have been well established (54), less is known about circulating proteins and additional signaling molecules. Acute bouts of exercise may lead to substantial changes in circulating proteins, metabolites, or microRNAs; however, these changes may be transient, may or may not lead to changes in the resting levels of individual plasma biochemicals after repetitive bouts of exercise, and, further, may or may not lead to directionally concordant changes (111–113). For example, IL-6 levels increase markedly during acute bouts of exercise, enhancing insulin-stimulated glucose uptake and fat oxidation and promoting an antiinflammatory milieu (106, 114, 115); however, elevated basal (or resting) levels of IL-6 have been widely associated with an increased risk of incident CVD and T2D (116–118), and limited human data suggest that basal levels remain largely unchanged after regular exercise (119, 120). Taken together, these data and others suggest a context-dependent effect of IL-6 in exercise (121), with its pulsatile increases during acute exercise — as opposed to gradual changes in its basal levels — conferring its beneficial effects. Other exerkines, such as cathepsin B (CTSB) and GLPD1, may require increased basal levels after cumulative bouts to subsequently impart physiologic effects (122–124). Ultimately, the kinetic, physiologic effects of individual exerkines are likely to vary among the heterogeneous collection of biochemicals.

### Modes of transport.

As described above, exerkines represent a diverse group of compounds that enter the circulation through different means. It has long been established that proteins with a secreted signal peptide sequence (“classically” secreted proteins) can be stimulated by exercise and released into the circulation and impart health effects (125). Similarly, small-molecule metabolites — including lactate — are readily transported into circulation by solute carriers (126). In contrast to these more well-established biologic processes, it is increasingly recognized that many proteins are secreted by nonclassical means (127). In particular, extracellular vesicles (EVs) have emerged as an important source of nonclassical protein secretion — as well as other bioactive cargo — in response to exercise. EVs are a heterogeneous (by size) group of lipid membrane–enclosed spheres that house biologic material, including proteins, DNAs, and RNA species, and transport it between cells (128). Recent studies have demonstrated substantial EV responsiveness to acute aerobic and resistance exercise bouts (129–131), highlighted a large number of EV proteins that are not annotated as classically secreted proteins (reviewed in ref. 125), and shown a broad range of EV tissue sources (132). While this topic has been reviewed elsewhere (133, 134), it represents an important reminder that much work remains to be done regarding our understanding of the plasma secretome.

### Exerkines and the cardiovascular system.

Exercise and increased physical activity provide protection against the development of CVD that extends well beyond that expected from their effects on traditional risk factors (135). Indeed, their effects on nontraditional risk factors such as improvements in autonomic nervous system and skeletal muscle health and endothelial function have been proposed as potentially filling these gaps (136, 137).

**Figure 3:**
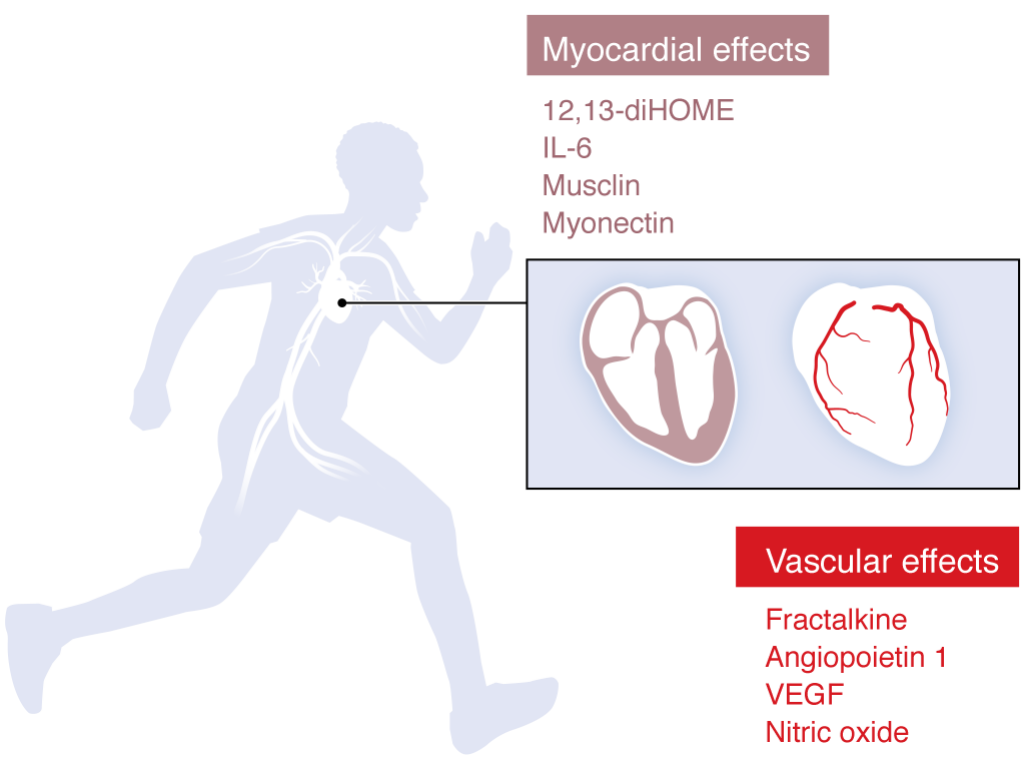
Cardiovascular-specific exerkines. Exercise-induced secreted and bioactive factors relevant in cardiovascular physiology. Fractalkine, angiopoietin-1, VEGF, and nitric oxide have effects on vascular biology, including the coronary arteries, whereas 12,13-dihydroxy-9Z-octadecenoic acid (12,13-diHOME), IL-6, musclin, and myonectin have direct effects on the myocardium.

The diverse effects of exerkines on metabolic and vascular health coupled with increasing data supporting interorgan crosstalk with the heart (138) support the potential for exerkines to play an important role in mediating cardiovascular wellness. In addition to the well-established roles of nitric oxide and vascular endothelial growth factor in endothelial function and angiogenesis in response to exercise (139–142), we highlight emerging data regarding relevant exerkines and the cardiovascular system (Figure 3). The following cardiovascular-specific exerkines represent a wide range of circulating chemicals, from small-molecule metabolites to immunologic proteins to natriuretic peptide–like muscle-derived hormones.

### 12,13-diHOME.

12,13-Dihydroxy-9Z-octadecenoic acid (12,13-diHOME) is an oxidized linoleic acid metabolite secreted by brown adipose tissue (BAT) that was recently shown to increase significantly in male and female human subjects after cold (14°C) exposure as well as in response to an acute bout of moderate-intensity exercise (143, 144). 12,13-diHOME is inversely associated with insulin sensitivity and BMI, and has been shown to enhance fatty acid uptake in both BAT and skeletal muscle. More recently, Pinckard and colleagues (145) extended their previous findings by implicating 12,13-diHOME’s favorable impact on the heart. Through a series of experiments including BAT transplantation in age- and sex-matched C57BL/6 mice, 8 weeks of interval-based treadmill training in mice, acute treatment with and sustained overexpression of 12,13-diHOME using tissue nanotransfection, and both echocardiography and invasive hemodynamics, the investigators showed that 12,13-diHOME led to favorable cardiac remodeling and improved ino- and lusitropy (i.e., cardiac contractility and relaxation) in mice. They further demonstrated that in addition to increasing fatty acid uptake in the heart (as previously shown in skeletal muscle), 12,13-diHOME enhanced cardiomyocyte respiration in a nitric oxide synthase type 1–dependent (NOS1-dependent) manner. And finally, plasma levels of 12,13-diHOME were lower in men and women with heart failure compared with healthy subjects. Taken together, these data support 12,13-diHOME as a lipokine that imparts direct, beneficial effects on the myocardium in an endocrine-like manner.

### Interleukin-6.

Perhaps the most well-recognized myokine, IL-6 is an important metabolic regulator in skeletal muscle whose exercise effects were briefly described above and have been extensively reviewed elsewhere (106, 121). More recently, a secondary analysis from a randomized controlled trial of a 12-week aerobic exercise training intervention with or without IL-6 antagonism (via monthly injections of tocilizumab) in 52 mostly female (79%) abdominally obese adults showed that IL-6 blockade led to significant attenuation in pericardial fat loss and physiologic hypertrophy as well as increases in epicardial fat mass (146). These data suggest an IL-6–dependent role for favorable cardiac adaptations to aerobic exercise training.

### Fractalkine.

Fractalkine (CX3CL1) is an additional immunologic protein and unique chemokine that is both membrane-bound and secreted into the circulation. Human studies have shown that acute bouts of endurance exercise lead to significant increases in both skeletal muscle mRNA and protein levels — mainly in endothelial cells — as well as circulating protein levels (147, 148). Fractalkine mediates survival in several cell lines, including monocytes, and has been shown to promote atherosclerotic lesion progression in *Apoe^–/–^* mouse models (149, 150). However, its role in atherogenesis may be context-dependent in regard to lesion development, and furthermore, fractalkine mediates smooth muscle cell recruitment and demonstrates antifibrotic effects in a liver injury model as well as tissue healing, all supporting a likely diverse role for fractalkine in vascular biology (151). Thus, additional study of the relationship between exercise-secreted fractalkine and its cardiovascular effects is warranted.

### Myonectin.

Murine studies of myonectin, a member of the C1q/TNF-related protein (CTRP) family, have shown that its skeletal muscle expression and circulating levels increase in response to regular aerobic exercise (152, 153). Using a combination of a mouse model with global myonectin knockout, a gain-of-function model in skeletal muscle, and in vitro cardiomyocyte studies, Otaka et al. demonstrated a crucial role for myonectin in attenuating ischemia/reperfusion injury through the sphingosine-1-phosphate (S1P)/cAMP/Akt (protein kinase B) signaling pathway in exercise-trained mice (153). More investigation is needed to understand myonectin’s mechanistic role in the myocardium, and whether it mediates cardioprotection in additional pathologic states.

### Musclin.

Musclin is a secreted peptide with high homology to the natriuretic peptides. In a series of in vivo murine studies and primary myoblast lines, Subbotina et al. (154) showed that skeletal muscle expression and circulating levels of musclin were greatly increased after 5 days of treadmill running (45 min/d) through a Ca^+2^/Akt/FOXO1 signaling pathway. Using a murine musclin (*Ostn*) knockout model, the authors then showed that *Ostn*-knockout mice had reduced exercise tolerance despite exhibiting an otherwise normal resting metabolic phenotype, and that musclin administration “rescued” their exercise capacity. *Ostn*-knockout mice demonstrated reduced VO_2_max after 5 days of training, an effect mediated by musclin’s potentiation of the effects of atrial natriuretic peptide (ANP) on cyclic guanosine monophosphate (cGMP)/PGC-1α regulation and subsequent mitochondrial biogenesis (154). More recently, investigators showed that *Ostn*-knockout mice demonstrate exaggerated cardiac dysfunction in a murine pressure overload (transverse aortic constriction) model, and that AAV6-mediated skeletal muscle musclin overexpression attenuates cardiac dysfunction and myocardial fibrosis in the same model (155). Mechanistically, musclin enhances cardiomyocyte contractility and prevents fibroblast activation through a C-type natriuretic peptide (CNP) signaling pathway. The authors then showed in a small human sample that serum musclin levels were significantly lower in patients with heart failure than in healthy controls. Taken together, these data support the potential of musclin as a relevant exerkine in cardiovascular health.

### Angiopoietin-1.

Angiopoietin-1 (ANG1) is a secreted member of the angiopoietin/TIE growth factor receptor pathway that mediates vascular protection through inhibition of vascular inflammation and plasma leakage and attenuates fibrosis (156). Its therapeutic potential is highlighted by promising preclinical studies of ANG1 ligands (157). Conflicting and limited data exist regarding the effect of both acute and chronic exercise on ANG1. *ANG1* skeletal muscle expression increased significantly in a group of sedentary male human subjects who demonstrated substantial improvements in CRF after 6 weeks of endurance exercise training (158); however, expression levels were mixed in heterogeneous skeletal muscle samples (fast-twitch white and red fibers and slow-twitch fibers) from Sprague-Dawley rats after regular aerobic exercise (159). Similarly, skeletal muscle expression of ANG1 was unchanged in a small (*N* = 7) cohort of young, sedentary male human participants after acute resistance exercise (160).

## Limitations and important questions remaining in the field

Despite the many advances in our understanding of the substrate metabolism involved in exercise performance and adaptation, as well as emerging knowledge of the whole organ circuitry involved, numerous gaps in the field remain (Table 1).

Well-executed human training studies have informed our understanding of exercise-induced cardiometabolic adaptations over the past few decades (83, 161–163); however, questions remain for nearly every human population (healthy and diseased) regarding the optimal type, duration, and intensity of exercise. What is the most beneficial exercise strategy for improving insulin and glucose homeostasis in individuals with impaired glucose tolerance compared with those with established T2D? How does the impact of weight loss affect cardiac performance among individuals with heart failure with preserved ejection fraction? How does predominant resistance training compare with endurance training in mediating improvements in VO_2_max? Many questions stem, in part, from intra- and interindividual differences in exercise training response according to clinical outcome (14, 164, 165). This underscores not only the potential opportunity for precision exercise medicine, but also the need for additional well-designed human exercise studies both for discovery and to validate existing findings that have been demonstrated in single studies or small samples.

Several barriers exist in the translation of findings from animal — and, in particular, rodent — exercise studies to humans. First, several promising findings from rodent studies have not been borne out in humans, as discussed previously, likely owing to a combination of environmental factors (i.e., training protocol differences, nutritional state), sex differences, and evolutionary biology. These concepts have been previously reviewed (166). Second, outside of the blood, there remains a lack of tissue sampling from exercising humans at scale to translate findings from model systems and preclinical animal studies.

Similarly, enthusiasm for findings regarding the role of exerkines in human health and disease is tempered by many questions. What are the effective concentrations of candidate exerkines needed to promote physiologic effects in humans? What are the temporal relationships of exerkines across acute exercise bouts compared with regular exercise? What are the specific tissue sources of exerkines, and how do they differ from the resting (basal) state?

These challenges highlight the rich opportunities to advance the field of exercise biology.

## Path forward: translating exercise biology into therapeutic advances

A convergence of factors, including increased appreciation for exercise’s pleiotropic health benefits, renewed commitment to its study, and the dissemination of high-throughput molecular profiling technologies, has led to a surge of new information in exercise biology. How to harness this information and translate findings into therapeutic advances remains an important challenge. Several approaches may help bridge this gap and inform future directions in the field (Table 1).

First, while much of the molecular biology work performed thus far has focused on single organ systems and/or used one, or possibly two, omics-based approaches, it is crucial to develop a molecular “road map” of exercise’s biochemical effects given the maturity of these technologies. Efforts to do so are ongoing, most notably in the NIH’s Molecular Transducers of Physical Activity Consortium (MoTrPAC) study, a multicenter, clinical trial of resistance and endurance exercise training that aims to generate such a road map across the plasma, skeletal muscle, and adipose tissue in sedentary and highly active human subjects, and across animal studies using genomics and epigenomics, proteomics and phosphoproteomics, metabolomics, and transcriptomics among other molecular profiling methods (7, 167). The cellular and molecular insights generated from such approaches must then be wedded to careful clinical physiologic phenotyping in healthy individuals as well as in those with disease states in future studies. The effort and resources involved in performing such work underscore the importance of making data readily available to the entire scientific community to help advance the field.

Second, connecting observations made in exercise intervention studies with longitudinal studies of human health to assess long-term outcomes can help triage findings. The example of exercise response biomarkers helps illustrate this concept. While far from ready for clinical adoption, several studies highlight the potential of using molecular biomarkers to predict an individual’s responsiveness to a given exercise intervention (i.e., “precision exercise medicine”) (168, 169). Most exercise training studies, however, are limited to short-term clinical outcomes (e.g., exercise adaptations) and/or relegated to small samples because of the complexity and cost of executing exercise trials. Here, leveraging large, population-based studies to demonstrate a candidate exercise biomarker’s relationship to long-term health outcomes can help provide an important layer of evidence for its relevance as a marker of human health and disease (170–172).

In the same vein, large human genetic studies may also provide opportunities to assess causality for an exercise-relevant biochemical or molecular pathway. As technological advances have enabled researchers to perform molecular profiling techniques at very large scale, Mendelian randomization experiments have been increasingly used to assess putative causal relationships between genetic variation, intermediate molecular phenotypes (i.e., circulating biochemicals), and health outcomes (173–177). These data sets may provide a valuable resource for the scientific community to triage an expanding list of exercise-relevant factors.

Finally, retro-translating human exercise findings to animal and model systems is important not only to generate mechanistic insights, but also to establish the tissue source of exerkines. Indeed, as discussed above, many of the known exerkines (e.g., adiponectin, FGF-21, myostatin) and undoubtedly many yet-to-be-identified exerkines are expressed in multiple tissue sources. While the study of extracellular vesicles in the transport of exercise-secreted proteins and microRNAs has provided important insights into their relevance (133), little is known about the specific tissue sources of most exercise-secreted factors. Finally, new techniques that use proximity labeling and allow for in vivo assessment of the spatial and temporal dynamics of circulating proteins offer promise toward unraveling the specific cell and tissue sources of these targets (178, 179).

## Conclusions

Our understanding of the organ- and even cell-specific activity involved in the cardiometabolic adaptations to exercise has evolved considerably over the past several decades, fueled by advances in molecular and cellular biology. These gains have helped reveal the extraordinary metabolic plasticity that enables such a broad spectrum of human locomotor activity — from short, maximal-intensity bursts of activity to ultra-endurance athletic events. While the skeletal muscle, liver, and adipose tissue remain central actors, the heart remains a crucial engine whose study may lead to novel therapeutic advances in cardiometabolic health and disease. Integrative multi-omics and systems biology approaches to studying exercise have helped unmask the complex whole-organism circuitry that is marshalled in response to physical activity, and offer promise to understand the systemic health benefits that this circuitry provides at the molecular level. Much work remains to be done in the field of exercise science, and the gaps that remain should be viewed as an enormous opportunity for the medical community. And finally, it remains important to emphasize the public health message that any physical activity is better than none while working toward a more complete understanding of the molecular and biochemical pathways that underlie exercise’s rich health benefits.

## Figures and Tables

**Table 1:**
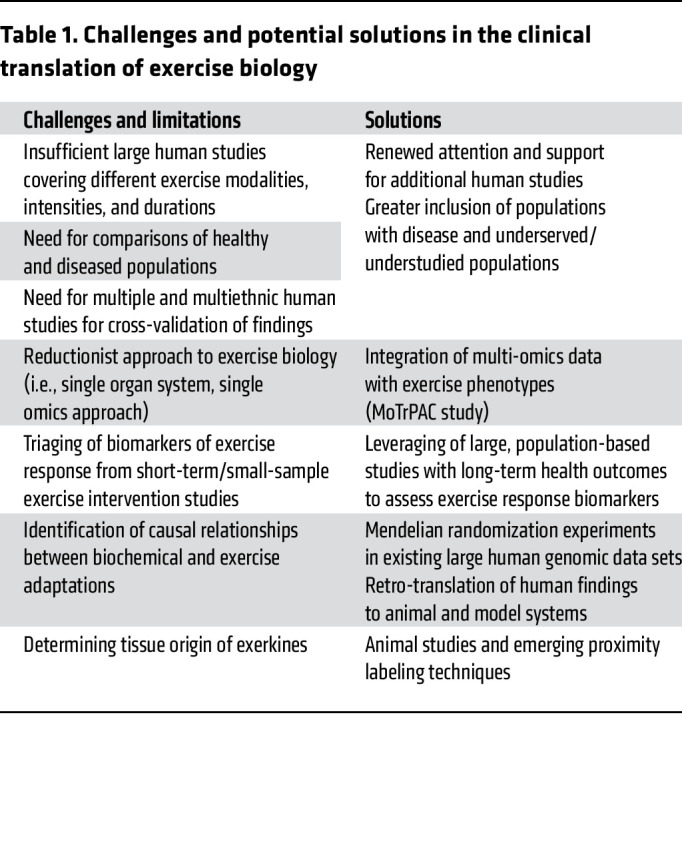
Challenges and potential solutions in the clinical translation of exercise biology

## Version 1 06/01/2023

Electronic publication
