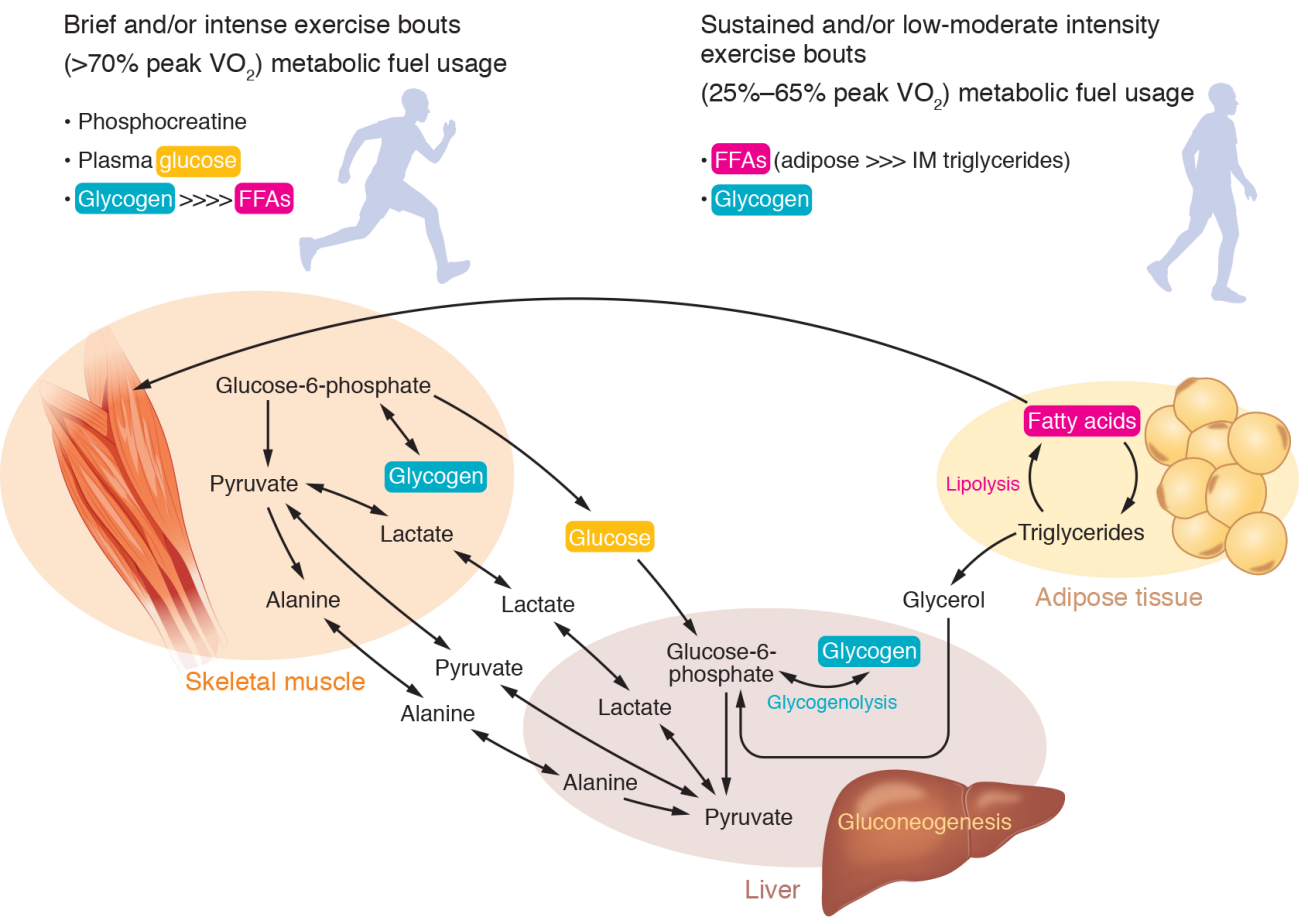# Exercise, exerkines, and cardiometabolic health: from individual players to a team sport

**DOI:** 10.1172/JCI172916

**Published:** 2023-07-03

**Authors:** Jeremy M. Robbins, Robert E. Gerszten

Original citation: *J Clin Invest*. 2023;133(11):e168121. https://doi.org/10.1172/JCI168121

Citation for this corrigendum: *J Clin Invest*. 2023;133(13):e172916. https://doi.org/10.1172/JCI172916

After the publication of this Review, the authors became aware of errors in Figure 1 related to details shown in the metabolic pathways and made several corrections to the figure. The correct figure is below.

## Figures and Tables

**Figure:**